# Supplementary material for: The interactome of intact mitochondria by cross-linking mass spectrometry provides evidence for coexisting respiratory supercomplexes
Source: Mol Cell Proteomics. 2017 Dec 8;17(2):216–32. doi: 10.1074/mcp.RA117.000470 (PMC5795388; doi:10.1074/mcp.RA117.000470)
Supplement: Supplemental Data [file supp_RA117.000470_133922_0_supp_23978_fzffwf.zip › spectra_annotation/mito_DR_spectra_annotation/18-1-1-1-8-1.pdf]

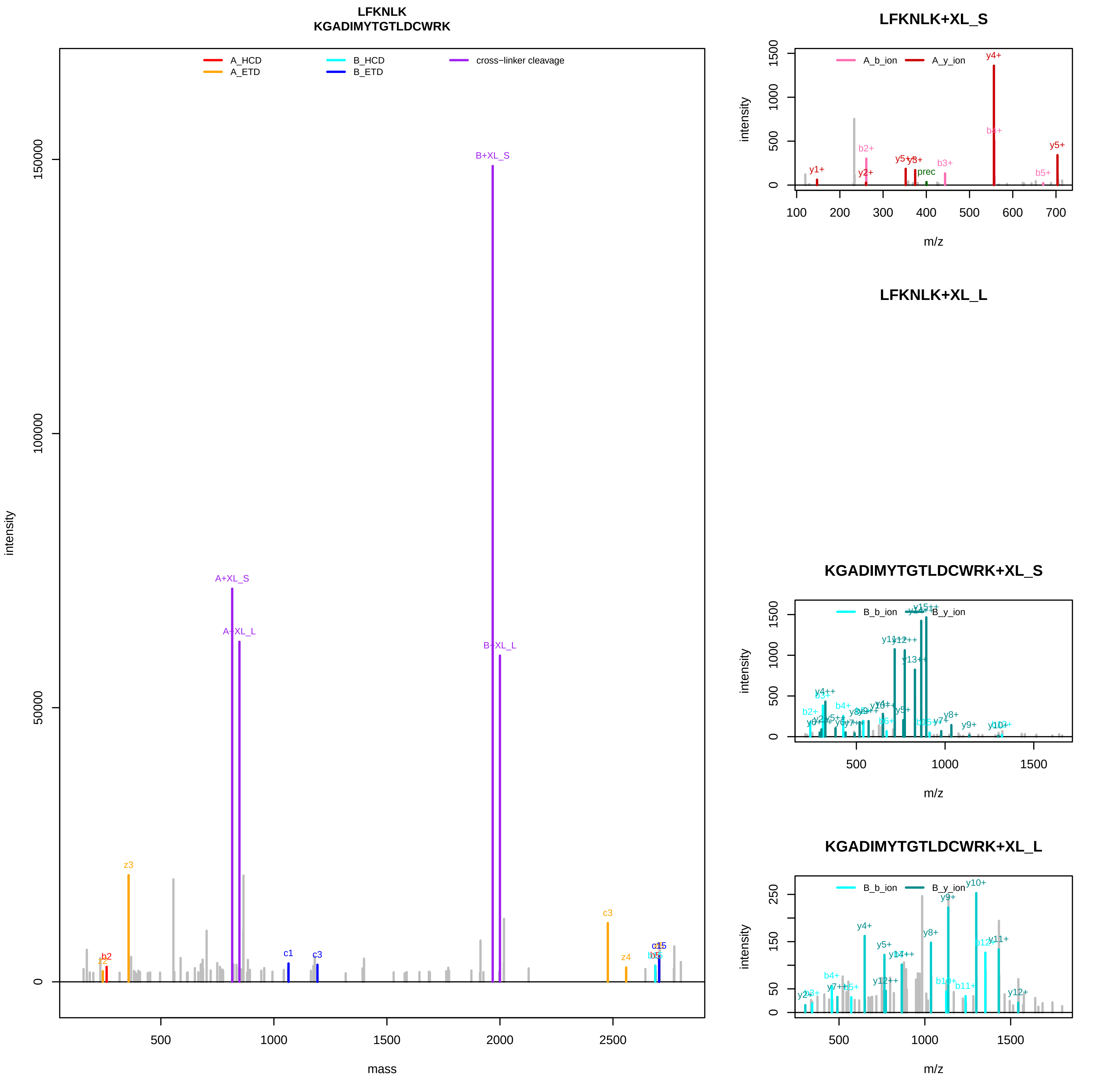

LFKNLK
KGADIMYTGTLDCWRK
A_HCD
A_ETD
B_HCD
B_ETD
cross−linker cleavage
B+XL_S
A+XL_S
A+XL_L
B+XL_L
z3
c3
z4
c1
c3
c15
b2
z2
intensity
mass
0
50000
100000
150000
500
1000
1500
2000
2500
LFKNLK+XL_S
A_b_ion
A_y_ion
y4+
b4+
y5+
b2+
y5++
y3+
b3+
y1+
y2+
prec
b5+
intensity
m/z
LFKNLK+XL_L
KGADIMYTGTLDCWRK+XL_S
B_b_ion
B_y_ion
y15++
y11++
y12++
y13++
y4++
b3+
b2+
b4+
y8+
y9+
y10+
intensity
m/z
KGADIMYTGTLDCWRK+XL_L
B_b_ion
B_y_ion
y10+
y9+
y4+
y8+
y5+
y14++
y11+
y12+
y12++
b4+
b10+
b11+
y2+
intensity
m/z
